# Serum miR-214 Serves as a Biomarker for Prodromal Parkinson’s Disease

**DOI:** 10.3389/fnagi.2021.700959

**Published:** 2021-10-28

**Authors:** Lanting Li, Jingru Ren, Chenxi Pan, Yuqian Li, Jianxia Xu, Hui Dong, Yong Chen, Weiguo Liu

**Affiliations:** ^1^Department of Neurology, The Affiliated Brain Hospital of Nanjing Medical University, Nanjing, China; ^2^Department of Medical Genetics and Cell Biology, School of Basic Medical Sciences, Zhengzhou University, Zhengzhou, China; ^3^Department of Laboratory, The Affiliated Brain Hospital of Nanjing Medical University, Nanjing, China

**Keywords:** Parkinson’s disease, prodromal, miR-214, serum, biomarker

## Abstract

Circulating microRNAs (miRNAs) have been proposed to be accessible biomarkers for Parkinson’s disease (PD). However, there is a lack of known miRNAs that can serve as biomarkers for prodromal PD (pPD). We previously identified that miR-31 and miR-214 were dysregulated in PD. The aim of this study was to explore the roles of miR-31 and miR-214 in pPD. We recruited 25 pPD patients, 20 patients with* de novo* PD (dnPD), 24 advanced PD (aPD) patients and 21 controls. Next, we investigated the expression of miR-31 and miR-214. Compared to controls, miR-214 was found to be significantly upregulated in pPD patients while miR-31 was significantly upregulated in aPD patients. In addition, the expression of miR-214 was lower in aPD patients compared to both dnPD or pPD patients, while the expression of miR-31 was higher in aPD patients compared to dnPD patients. In order to predict pPD *via* miRNA expression, the receiver operating characteristic curve was constructed and the area under curve (AUC) was calculated. For pPD prediction by miR-214, the AUC was 0.756. The optimal cut-off value of miR-214 was 0.1962, and the sensitivity and specificity were 72.0% and 76.2%, respectively. On the other hand, the AUC for aPD detection by miR-31 was 0.744. The optimal cut-off value for miR-31 was 0.0148, with a sensitivity of 87.5% and a specificity of 71.4%. In conclusion, miR-214 can distinguish pPD patients from controls and may be used as a potential biomarker for pPD diagnosis.

## Introduction

The diagnosis of Parkinson’s disease (PD) is mainly based on classical motor symptoms, including bradykinesia, rigidity, rest tremor, and postural instability (Kalia and Lang, 2015). Pathologically, approximately 50–70% of dopaminergic neurons in the substantia nigra pars compacta (SNpc) have already degenerated by the time patients develop motor symptoms and a diagnosis of PD can be made (Grosset and Schapira, 2008). However, various non-motor symptoms are known to precede motor symptoms by years (Gaenslen et al., 2011; Pont-Sunyer et al., 2015; Schapira et al., 2017). The period during which non-motor and/or mild motor symptoms occur is been referred to as the prodromal stage of PD (Gaenslen et al., 2014; Mahlknecht et al., 2015; Marsili et al., 2018). Exploring the prodromal stage of PD is important for the identification of the disease at its early stage.

Prodromal PD (pPD) is characterized by the presence of early symptoms and signs of PD neurodegeneration that do not fulfill the criteria for clinical diagnosis. The International Parkinson and Movement Disorder Society (MDS) has published research criteria for pPD (Berg et al., 2015; Heinzel et al., 2019). The criteria are based on the likelihood of pPD being present, defining possible and probable pPD as having a probability score of 30% to 80% and ≥80%, respectively. The probabilities of pPD for individuals can be calculated from the predictive information of risk and prodromal markers using a web-based pPD risk calculator. Olfactory dysfunction, rapid eye movement (REM) sleep behavior disorder (RBD), constipation, depression, and cognitive impairment are all prodromal markers (Berg et al., 2015; Heinzel et al., 2019). Individuals with prodromal markers are at higher risk for developing PD in the future, and developing potential markers for the prodromal phase can help improve the diagnostic accuracy of pPD.

MicroRNAs (miRNAs) are small non-coding RNAs that function as post-transcriptional regulators of gene expression. They bind to the complementary sequence of their target messenger RNAs (mRNAs), which leads to either translational inhibition or degradation of the mRNAs (Makeyev and Maniatis, 2008; Bartel, 2009; Ghildiyal and Zamore, 2009). Aberrantly expressed miRNAs are known to contribute to several neurodegenerative diseases, including PD (Junn and Mouradian, 2012; Lu et al., 2017; Goh et al., 2019; Juźwik et al., 2019). Ongoing studies have demonstrated that miRNAs are suitable biomarkers for PD (Khodadadian et al., 2018; Roser et al., 2018; Barbagallo et al., 2020). Additionally, serum has been widely used in biomarker studies due to its non-invasiveness and easy accessibility. Moreover, dysregulated miRNAs play a role across the PD timeline, ranging from the non-motor to the overt motor phase (Titze-de-Almeida et al., 2021). The findings in RBD patients have suggested that the abnormality of serum miRNAs is associated with pPD (Fernández-Santiago et al., 2015). This provides the possibility that serum miRNA profiling can be a novel strategy for diagnosing pPD.

To the best of our knowledge, there is a lack of miRNAs that can serve as biomarkers for pPD diagnosis. Developing biomarkers for an early diagnosis of PD can help delay the development and progression of the disease. Therefore, it is highly imperative to explore biomarkers that can help make an accurate diagnosis at an early stage of PD. In our previous study, we identified that miR-31 and miR-214 were dysregulated in PD (Dong et al., 2016; Yan et al., 2020). In order to further explore their roles in the prodromal phase of PD, we investigated the expression of these two miRNAs in the serum samples from patients with pPD. In addition, we quantified the miRNAs expression in *de novo* PD (dnPD) patients at an early stage and the advanced PD (aPD) patients in order to clarify whether expression of miRNAs is associated with disease severity.

## Materials and Methods

### Subjects

In total, 90 subjects were enrolled, which included 25 pPD patients, 20 dnPD patients, 24 aPD patients, and 21 normal controls (NC). Patients with dnPD and aPD were recruited from the Movement Disorder Clinic at the Department of Neurology of Brain Hospital affiliated with the Nanjing Medical University. The inclusion criteria for dnPD were as follows: (1) The patient must be newly diagnosed with PD according to the United Kingdom Parkinson’s Disease Society Brain Bank clinical diagnostic criteria (Gibb and Lees, 1988); (2) not have been treated yet; (3) have more than a 30% improvement rate in the Unified Parkinson’s Disease Rating Scale (UPDRS) part III by the standard acute levodopa challenge test; (4) have early-stage PD (modified Hoehn and Yahr (H-Y) ≤2); and (5) have had a follow-up through hospital visits for at least 1 year. The inclusion criteria of aPD were as follows: (1) The patient must be diagnosed with PD based on the United Kingdom Parkinson’s Disease Society Brain Bank clinical diagnostic criteria (Gibb and Lees, 1988); (2) have a beneficial response to levodopa; and (3) have late-stage PD (modified H-Y >2). The exclusion criteria for both dnPD and aPD included: (1) the presence of atypical or secondary Parkinsonism; (2) obvious clinically significant lesions on brain magnetic resonance imaging (MRI) scans; (3) severe chronic diseases, such as severe psychiatric and systemic illnesses, heart failure, diabetes and cancer; and (4) difficulty in completing the clinical assessment.

Patients with pPD were recruited from a community-based prospective study of pPD patients that was conducted at the Department of Neurology of Brain Hospital affiliated with the Nanjing Medical University. A standardized structured questionnaire was utilized to screen pPD in the community, which included occupational solvent exposure, regular pesticide exposure, family history, coffee or tea use, smoking status, olfactory loss, substantia nigra (SN) hyperechogenicity, RBD, and possible subthreshold Parkinsonism. Then, individuals with possible/probable pPD were examined through a hospital visit by two specialized neurologists. Patients were diagnosed with pPD based on MDS research criteria for pPD, in which patients were diagnosed if the probability of pPD ≥80% (Berg et al., 2015). The exclusion criteria included: (1) a diagnosis of PD, atypical or secondary parkinsonism; (2) the presence of other neurological disorders; (3) serious chronic diseases, such as severe psychiatric and systemic illnesses, heart failure, diabetes and cancer; and (4) difficulty in completing the clinical assessment. The controls matched to patients in gender and age were recruited from the affiliated Brain Hospital of Nanjing Medical University.

The study was approved by the ethics committee of the Second Affiliated Hospital of Zhejiang University School of Medicine and the Affiliated Brain Hospital of Nanjing Medical University. Written informed consent was obtained from all participants. Additionally, venous blood samples were obtained from all participants. Serum was extracted from whole blood after centrifugation (15 min at 3,000 *g*) and stored at −80°C until further processing.

### Quantitative Real-Time Polymerase Chain Reaction (qRT-PCR) Assay of Serum miRNAs

Total RNA was extracted utilizing the miRcute Serum/Plasma miRNA Isolation Kit (#DP503, TIANGEN, Beijing, China). Next, the RNA was reverse transcribed to cDNA using the miRcute Plus miRNA First-Strand cDNA Kit (#KR211, TIANGEN, Beijing, China), as per the manufacturer’s instructions. A qRT-PCR assay was performed using miRcute Plus miRNA qPCR Kit (SYBR Green; #FP411, TIANGEN, Beijing, China) on the 7300 Sequence Detection System (Applied Biosystems, Foster, USA). For each sample, qRT-PCR was carried out in triplicate. The expression of the target miRNAs was normalized to the level of external reference (TIANGEN, Beijing, China). The relative quantification of miRNA was calculated using the 2^−△Ct^ method.

### Clinical Assessment

Demographic characteristics of participants were recorded, including age, gender, age at onset, and duration of disease. PD patients were evaluated by the UPDRS part I, II, III, IV, as well as modified H-Y stages. Non-motor symptoms were quantified using the Non-Motor Symptoms Questionnaire (NMSQ). The Mini-Mental State Examination (MMSE) and Montreal Cognitive Assessment (MoCA) were utilized to assess cognition. Depression and anxiety were evaluated using the Hamilton Depression Rating Scale (HAMD) and Hamilton Anxiety Scale (HAMA). Sleep was evaluated with the Parkinson’s Disease Sleep Scale (PDSS) or REM Sleep Behavior Disorder Questionnaire-Hong Kong (RBDQ-HK; Shen et al., 2014).

### Statistical Analysis

Statistical analysis was conducted using SPSS Statistics Version 24. Data were presented as median and 25th–75th percentile range, with the exception of gender category. A nonparametric Mann-Whitney *U* test was used to analyzd the differences between two groups. Differences in gender between groups were compared utilizing the chi-square test. The diagnostic value of serum miRNAs as biomarkers was assessed by receiver operating characteristic (ROC) curve and the area under curve (AUC) was calculated. The optimal cut-off value of miRNAs was determined by Youden’ method. For diagnostic tests, sensitivity, specificity, positive predictive value, and negative predictive value were used as performance measures. The relationship between the expression of miRNAs and clinical characteristics was determined using Spearman’s rank correlation analysis. *P* < 0.05 was considered to be significant.

## Results

### Comparison of miR-31 and miR-214 Expression Across Different Groups

**Figure 1:**
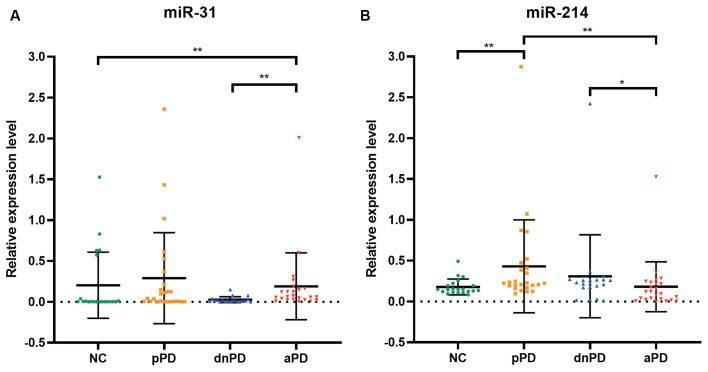
The expression of miR-31 **(A)**, miR-214 **(B)** in each group. *P < 0.05, **P < 0.01.

The expression of miR-31 and miR-214 were investigated in 90 subjects, including 25 pPD patients, 20 dnPD patients, 24 aPD patients, and 21 NC (Table 1). There were no intergroup differences with regards to age and gender (*P* > 0.05). The expression of miR-31 in the serum was found to be significantly higher in the aPD group compared to the NC group (*P* = 0.005, Figure 1A). Furthermore, there was no significant difference between NC and pPD group or dnPD group (*P* > 0.05). These results suggest that miR-31 may function as a biomarker for aPD. In addition, serum miR-31 expression was higher in the aPD group, compared to the dnPD group (*P* = 0.001, Figure 1A), which suggests miR-31 levels tend to increase with disease severity.

**Table 1:** Baseline characteristics of the patients in each group.

**Variables**	**NC (*n* = 21)**	**pPD (*n* = 25)**	**dnPD (*n* = 20)**	**aPD (*n* = 24)**
Age (years)	64.00 (62.00–66.00)	68.00 (63.00–70.00)	65.00 (64.00–68.00)	66.50 (63.25–69.00)
Gender ratio (F/M)	11/10	12/13	11/9	12/12
Age at onset (years)	NA	NA	64.00 (61.25–66.75)	58.00 (54.25–61.00)
Disease duration (years)	NA	NA	1.50 (1.00–2.75)	8.00 (7.00–9.00)
UPDRS I	NA	NA	4.00 (3.00–5.75)	5.50 (3.25–7.00)
UPDRS II	NA	NA	8.00 (6.00–12.75)	15.00 (11.25–18.75)
UPDRS III	NA	6.00 (4.00–7.50)	23.00 (20.25–37.75)	39.00 (31.25–48.00)
UPDRS IV	NA	NA	0.00 (0.00–0.00)	0.50 (0.00–1.75)
H-Y	NA	NA	2.00 (1.13–2.00)	2.50 (2.50–3.00)
NMSQ	NA	10.00 (5.50–15.00)	10.00 (5.25–13.75)	13.00 (10.00–17.00)
MMSE	30.00 (29.00–30.00)	28.00 (26.00–29.00)	27.00 (21.50–29.00)	27.50 (23.00–29.00)
MoCA	27.00 (25.00–28.00)	25.00 (21.50–26.00)	20.50 (15.75–23.00)	22.00 (14.50–24.75)
HAMD	1.00 (0.00–4.50)	7.00 (3.00–12.50)	12.00 (8.00–13.00)	15.00 (8.50–20.00)
HAMA	2.00 (0.00–4.50)	5.00 (1.50–10.50)	8.00 (4.00–10.75)	11.50 (6.25–13.00)
RBDQ-HK	NA	32.00 (18.50–44.50)	NA	NA
PDSS	NA	NA	124.50 (109.75–139.75)	106.00 (82.00–122.25)

*Values were expressed as the median and 25th–75th percentile range except for the gender category. NC, normal controls; pPD, prodromal Parkinson’s Disease; dnPD, *de novo* Parkinson’s Disease; aPD, advanced Parkinson’s Disease; UPDRS, Unified Parkinson’s Disease Rating Scale; H-Y, Hoehn and Yahr; NMSQ, Non-Motor Symptoms Questionnaire; MMSE, Mini-Mental State Examination; MoCA, Montreal Cognitive Assessment; HAMD, Hamilton Depression Rating Scale; HAMA, Hamilton Anxiety Scale; PDSS, Parkinson’s Disease Sleep Scale; RBDQ-HK, REM Sleep Behavior Disorder Questionnaire-Hong Kong; NA, not available*.

In comparison to the NC group, miR-214 was found to be remarkably higher in the pPD group (*P* = 0.003, Figure 1B). On the other hand, there was no difference between the NC and dnPD group or aPD group (*P* > 0.05). Furthermore, patients with aPD had lower miR-214 levels compared to patients with dnPD or pPD (*P* = 0.046 vs. 0.001, Figure 1B). Therefore, miR-214 can serve as a biomarker for pPD and has a tendency to decrease with disease severity.

### ROC Curve Analysis for Diagnosis of aPD or pPD

**Figure 2:**
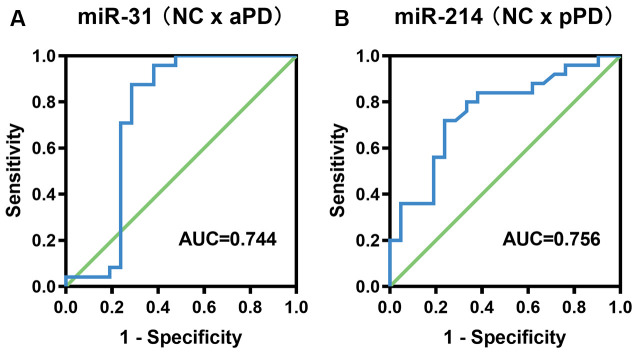
ROC curve analysis for the diagnosis of aPD **(A)**, pPD **(B)**. aPD, advanced Parkinson’s disease, pPD, prodromal Parkinson’s disease.

In order to evaluate the diagnostic accuracy of miR-31 and miR-214 as biomarkers, we constructed ROC curves. Analysis of a diagnosis of aPD demonstrated that the AUC was 0.744 (95% CI = 0.572–0.916, *P* = 0.005, Figure 2A). The optimal cut-off value of miR-31 was 0.0148, with a sensitivity of 87.5% and a specificity of 71.4%. The positive predictive value and negative predictive value using a miR-31 cut-off of 0.0148 were 77.8% and 83.3%, respectively. For pPD detection, the AUC was 0.756 (95% CI = 0.614–0.898, *P* = 0.003, Figure 2B). Additionally, the optimal cut-off value of miR-214 was 0.1962, with the sensitivity and specificity being 72.0% and 76.2%, respectively. The positive predictive value and negative predictive value using a miR-214 cut-off of 0.1962 were 78.3% and 69.6%, respectively.

### Correlation Analysis of miR-31 and miR-214 Expression and Clinical Characteristics

We carried out correlation analysis to determine whether miRNAs expression was related to clinical features. The dnPD and aPD group were combined for correlation analysis. The data indicated that age at onset was positively correlated with miR-214, and negatively correlated with miR-31 (Table 2). Moreover, miR-31 was positively correlated with disease duration, UP II score, UP III score, H-Y stage, HAMD score, while it was negatively correlated with PDSS score (Table 2). We also discovered that miR-214 may be negatively correlated with H-Y stage (*P* = 0.060). Finally, we found no significant association between miRNAs expression and clinical features in pPD patients (Table 3).

**Table 2:** Associations between serum miRNAs and clinical characteristics in dnPD and aPD patients by Spearman’s rank correlation analysis.

**Variables**	**miR-31**	**miR-214**
Gender	−0.088	0.023
Age	0.063	0.079
Age at onset	−0.409**	0.300*
Disease duration	0.553**	−0.221
UPDRS I	−0.001	−0.094
UPDRS II	0.321*	−0.151
UPDRS III	0.422**	−0.140
UPDRS IV	0.156	−0.108
H-Y	0.517**	−0.286
NMSQ	0.245	−0.060
MMSE	0.236	−0.034
MoCA	0.184	0.057
HAMD	0.310*	−0.144
HAMA	0.221	0.019
PDSS	−0.402**	0.143

UPDRS, Unified Parkinson’s Disease Rating Scale; H-Y, Hoehn and Yahr; NMSQ, Non-Motor Symptoms Questionnaire; MMSE, Mini-Mental State Examination; MoCA, Montreal Cognitive Assessment; HAMD, Hamilton Depression Rating Scale; HAMA, Hamilton Anxiety Scale; PDSS, Parkinson’s Disease Sleep Scale. *Represented correlation is significant at the 0.05 level (2-tailed). **Represented correlation is significant at the 0.01 level (2-tailed).

**Table 3:** Associations between serum miRNAs and clinical characteristics in pPD patients by Spearman’s rank correlation analysis.

**Variables**	**miR-31**	**miR-214**
Gender	0.394	0.205
Age	0.283	0.168
UPDRS III	−0.281	−0.156
NMSQ	0.087	−0.073
MMSE	−0.042	0.097
MoCA	0.133	0.198
HAMD	0.120	−0.114
HAMA	0.209	0.063
RBDQ-HK	0.240	0.312

*UPDRS, Unified Parkinson’s Disease Rating Scale; NMSQ, Non-Motor Symptoms Questionnaire; MMSE, Mini-Mental State Examination; MoCA, Montreal Cognitive Assessment; HAMD, Hamilton Depression Rating Scale; HAMA, Hamilton Anxiety Scale; RBDQ-HK, REM Sleep Behavior Disorder Questionnaire-Hong Kong*.

## Discussion

Herein, we investigated miRNAs expression in serum samples from controls, pPD, dnPD, and aPD patients using qRT-PCR assay. The results demonstrated that miR-214 was significantly upregulated in pPD patients and miR-31 was significantly upregulated in aPD patients compared to controls. The AUC for aPD detection by miR-31 was 0.744 (95% CI = 0.572–0.916), while the AUC for pPD detection by miR-214 was 0.756 (95% CI = 0.614–0.898). These findings suggest that miR-31 may function as a biomarker for aPD, while miR-214 may function as a biomarker for pPD. We also discovered that the expression of miR-31 was higher in aPD patients compared to dnPD patients, while the expression of miR-214 was lower in aPD patients compared to dnPD or pPD patients. Also, correlation analysis indicated that miR-31 was positively correlated with H-Y stage, while miR-214 was negatively correlated with H-Y stage. Furthermore, miR-31 increased with worse severity of non-motor and motor symptoms. These results demonstrate that miR-31 increases with disease severity, while miR-214 decreases with disease severity.

MiRNAs are abundantly expressed in the brain and exert diverse roles in the central nervous system (Meza-Sosa et al., 2012; Barry, 2014; Chen and Qin, 2015; Nowakowski et al., 2018). Accumulating evidence suggests that miRNAs contribute to many neuronal processes, including neurogenesis, differentiation, and development (Makeyev et al., 2007; Radhakrishnan and Alwin Prem Anand, 2016; Zainal Abidin et al., 2019). Thus, miRNAs likely have a critical function in the pathogenesis of PD. Circulating miRNAs have been proposed as potential biomarkers for early PD. A previous study disclosed that miR-4639 in plasma was significantly upregulated in early PD patients, with the H-Y stages of 1–2.5 or disease duration ≤2 years (Chen et al., 2017). Among early PD patients that were stratified in H-Y stages 1–2, miR-29a, miR-29c, and miR-19b were shown to be downregulated in the serum (Botta-Orfila et al., 2014). Similarly, miR-19b in serum was downregulated in RBD patients prior to clinical conversion to PD (Fernández-Santiago et al., 2015).

Herein, we demonstrated that miR-214 can serve as a potential biomarker for PD at the prodromal stage. Altered expression of miR-214 has previously been implicated in several neurological diseases, including Alzheimer’s disease (AD), multiple sclerosis (MS), and Huntington’s disease (Junker et al., 2009; Bekris et al., 2013; Hoss et al., 2015; Viswambharan et al., 2017). There have been reports on the relationship between miR-214 and PD, which have shown that serum miR-214 was downregulated in PD (Ma et al., 2016). Furthermore, in a previous study, we reported that miR-214 in serum was upregulated in early PD, and further downregulated as the disease progresses (Yan et al., 2020). This may be due to a compensatory response in the body at the early stage of PD. Herein, we discovered that miR-214 increases in the prodromal stage of PD. Besides, miR-214 has a tendency to decrease with disease severity. The evidence further strengthened our hypothesis. However, there were no significant differences in miR-214 expression between dnPD patients and controls. Both studies had a small sample size, and therefore, the results need to be validated in a larger sample size.

The mechanism of miR-214 in PD has been elucidated in previous studies. MiR-214 regulates the expression of α-synuclein, which is closely related to the pathogenesis of PD (Wang et al., 2015). The overexpression of miR-214 leads to a decrease in α-synuclein expression, which is a potential mechanism that underlies the neuroprotection in Resveratrol, a potential therapeutic drug in PD treatment. Another study demonstrated that miR-214 targets *KLF4* in order to alleviate 1-methyl-4-phenylpyridinium (MPP^+^)-triggered cytotoxicity in PD (Zhou et al., 2020).

MiR-31 has previously been reported to act as a biomarker for neurological disorders. A study on AD patients illuminated that miR-31 expression in the serum of AD patients was significantly decreased (Dong et al., 2015). However, no difference was observed between controls and patients with early AD. Upregulation of miR-31 was shown to be present in the serum of patients with multiple system atrophy (Kume et al., 2018). Additionally, the expression of miR-31 in peripheral blood mononuclear cells was lower in MS patients, compared to controls (Martinelli-Boneschi et al., 2012). Previously, we reported that miR-31 was markedly decreased in PD patients (Dong et al., 2016). Herein, we demonstrated that miR-31 was significantly higher in aPD patients compared to controls. The reason for this difference may be that patients involved in the previous study have both early-stage and late-stage PD. In addition, PD patients have a shorter disease duration with an average of 6 years. We also found that miR-31 was positively correlated with disease duration and H-Y stage, which can help explain the different results. The complicated role of miR-31 in PD pathogenesis may also contribute to diverse outcomes. It is well-known that apoptosis plays a major role in the neuronal death of PD (Lev et al., 2003; Tatton et al., 2003). miRNAs are involved in apoptosis by regulating a number of target genes and signaling pathways. Additionally, miR-31 has been shown to regulate apoptosis in various diseases, especially in cancers (Dong et al., 2014; Zhang et al., 2019). Furthermore, miR-31 is related to apoptotic regulation in neurological diseases. In spinal cord injury, miR-31 regulates apoptosis by mediating the PI3K/AKT signaling pathway (Wang et al., 2019). Therefore, we hypothesize that miR-31 may exert an effect on the regulation of apoptosis in PD.

Our study has several limitations. First, it is unknown whether pPD patients will eventually develop PD, as the patients were not followed up. In order to better clarify the alteration of miRNAs, the follow-up study of pPD patients is underway. Second, the results need to be validated in a larger cohort. The small sample size of our study was due to the difficulty in the enrollment of pPD patients. Patients with pPD were recruited from community-dwelling older people in Nanjing and the prevalence of pPD in an elderly population was only 2%. The recruitment of pPD patients has been in progress. Finally, we only investigated the expression of two miRNAs. It is plausible that other miRNAs may also play a role in the prodromal stage of PD.

In summary, we identified miR-214 in serum was upregulated in patients with pPD. The results indicated that miR-214 may be a potential biomarker for pPD and can help identify PD at an early stage. In our future research, we intend to construct a cellular model or a mouse model of PD to explore the underlying mechanism of miR-214 in PD.

## Data Availability Statement

The original contributions presented in the study are included in the article, further inquiries can be directed to the corresponding author.

## Ethics Statement

The studies involving human participants were reviewed and approved by the ethics committee of the Second Affiliated Hospital of Zhejiang University School of Medicine and the Affiliated Brain Hospital of Nanjing Medical University. The patients/participants provided their written informed consent to participate in this study.

## Author Contributions

WL designed this study and revised the manuscript. JR, CP, YL, and JX were responsible for data collection. LL, HD, and YC performed the experiment and analyzed the data. LL wrote the manuscript. All authors contributed to the article and approved the submitted version.

## Conflict of Interest

The authors declare that the research was conducted in the absence of any commercial or financial relationships that could be construed as a potential conflict of interest.

## Publisher’s Note

All claims expressed in this article are solely those of the authors and do not necessarily represent those of their affiliated organizations, or those of the publisher, the editors and the reviewers. Any product that may be evaluated in this article, or claim that may be made by its manufacturer, is not guaranteed or endorsed by the publisher.
